# Treatment Outcomes of Maxillary Sinus Squamous Cell Carcinoma at a Dedicated Cancer Institute: A Retrospective Study

**DOI:** 10.7759/cureus.25644

**Published:** 2022-06-03

**Authors:** Ahmed A Keerio, Muhammad U Qayyum, Alina Kashif, Rahim Dhanani, Asma Rashid, Muhammad Faisal, Raza Hussain, Arif Jamshed

**Affiliations:** 1 Otolaryngology, Shaukat Khanum Memorial Cancer Hospital and Research Centre, Lahore, PAK; 2 Surgical Oncology, Shaukat Khanum Memorial Cancer Hospital and Research Centre, Lahore, PAK; 3 Internal Medicine, Shaukat Khanum Memorial Cancer Hospital and Research Centre, Lahore, PAK; 4 Otolaryngology, Ziauddin University, Karachi, PAK; 5 Radiation Oncology, Shaukat Khanum Memorial Cancer Hospital and Research Centre, Lahore, PAK

**Keywords:** maxillary sinus, treatment outcome, prognosis, maxillary sinus neoplasms, squamous cell carcinoma

## Abstract

Introduction

Squamous cell carcinoma arising at the maxillary sinus is a rare neoplasm, characterized by an aggressive growth pattern and glooming prognosis. The proximity of the maxillary sinus with complex anatomical structures such as the eye, skull base, infratemporal fossa, pterygomaxillary fossa, nasal cavities, and ethmoid sinuses makes the surgical treatment of tumors infiltrating into these structures very challenging. The study's objective was to investigate the prognostic factors of survival and maxillary sinus SCC treatment outcomes.

Methods

We did a retrospective analysis of patients treated for maxillary sinus SCC at our institution between 2004 -2018. The study included all the patients with histologically proven maxillary sinus SCC treated with curative intent. The medical record of 43 patients was reviewed and utilized for the analysis. The Kaplan-Meier curve calculated five-year overall survival.

Results

A total of 43 patients were analyzed. At the presentation time, the mean age was 54.56 years (SD ± 11.65). Smoking (n=13, 30.2%) was the common risk factor. 36 (83.7%) patients presented with stage IV disease. Surgery was performed in 16 (37.2%) patients, whereas 27 (62.8%) patients received radiation and chemotherapy. Treatment failure was seen in 35 (81.4%) patients with locoregional recurrence in 30 (85.7%) patients and distant metastases in 5 (14.3%) patients. The five years overall survival in our study was 22%. Loco-regional recurrence and distant metastasis were the significant factors impacting survival (p=0.01).

Conclusion

Maxillary sinus SCC is rare cancer that is more common in males and usually presents at an advanced stage with a poor outcome. These tumors have a higher rate of treatment failure with a poor prognosis. Locoregional recurrence and distant metastasis adversely impact the overall survival.

## Introduction

The malignancies of the nasal cavity and paranasal sinuses are rare, with an annual occurrence of 1 per 100,000 populations [1-3]. The incidence of paranasal sinus tumors is 0.2-0.8%, with 60% of these tumors arising from the maxillary sinus, representing 3-5% of all malignancies of the head and neck region [1,4,5]. Maxillary sinus Squamous Cell Carcinoma (SCC) makes up 1 % of all malignancies. SCC remains the most prevailing malignancy regarding histopathology and makes up 80%. Other histologies include adenoid cystic carcinoma, sinonasal carcinoma, and adenocarcinoma. It is more common in males with a ratio of 2.3:1 and is common among the 50-60-year-old age group [1,2,6].

The proximity of the maxillary sinus with complex anatomical structures such as the eye, skull base, infratemporal fossa, pterygomaxillary fossa, nasal cavities, and ethmoid sinuses makes the surgical treatment of tumors infiltrating into these structures very challenging [7-9]. The most common etiological factors of maxillary sinus SCC are alcohol and smoking; they have synergistic effects like other head and neck sites. Other risk factors include wood dust and nickel exposure. Another challenge is the presence of nonspecific and vague symptoms, which can be mistaken for allergic, inflammatory, and infective causes. This is the reason for delayed diagnosis and hence an advanced stage in maxillary sinus malignancies [1,2,10]-most of the maxillary sinus malignancies present at stages III and IV. Common symptoms are blood-stained discharge, non-healing lesion, facial swelling, difficulty opening the mouth, and unilateral nasal obstruction. The proximity of the maxillary sinus with complex anatomical structures makes it difficult to diagnose the primary site of the tumor [11,12]. The lymph node metastasis is not commonly seen in squamous cell carcinoma of the maxillary sinus, and the incidence can range from 3.3-26% [1,2].

Maxillary sinus tumors that present at an early stage can be surgically treated with endoscopic resection. The advanced stage tumors require open surgical approaches that include lateral rhinotomy, maxillectomy, midfacial degloving, orbital exenteration, and craniofacial resection [13,14]. One of the biggest challenges in resectioning these tumors is achieving negative surgical margins [15,16]. Adjuvant treatment modalities like radiotherapy and chemotherapy are utilized in patients with involved margins. There is insufficient data on the treatment protocols, prognosis, and survival outcomes because of the limited number of cases compared to other areas of the head and neck region. Our study aims at identifying the predictors of survival and treatment outcomes of maxillary sinus SCC.

## Materials and methods

All the charts of the patients who presented with SCC of maxillary sinus treated at our center from January 2004 to December 2018 were retrospectively reviewed. Exemption from the ethical review committee was sought before data collection (IRB number EX-01-07-21-01, date of IRB July 30, 2021). All the patients with SCC of the maxillary sinus treated with curative intent were included in the study. Absconded cases or those who had other histological malignancies, inadequate data, incomplete treatment, or distant metastasis at the time of presentation, along with patients on palliative care, were excluded from the study to eliminate the bias and confounders.

Once the inclusion and exclusion criteria were established, we reviewed the medical records of the 43 patients counted in our study as primary variables were included in age, gender, risk factors, tumor staging, nodal status, margins status, and treatment failure. The data were analyzed with SPSS version 28.0. A p-value of < 0.05 was considered significant. The overall survival (OS) was calculated using the Kaplan-Meier curve.

## Results

Seventy-seven patients were diagnosed with SCC of the maxillary sinus and treated at our institute for 14 years (2004-2018). Out of these, 34 patients were excluded due to palliative intent, missing data, incomplete treatment, and loss of follow-up.

In our study, the disease predilection is slightly more common in males than female males (male to female ratio=1.5:1). At the presentation time, the mean age was 54.56 years (SD ± 11.65), ranging from 34 to 81 years. Smoking (n=13, 30.2%) was the common risk factor seen in our patients, whereas 16 (37.7%) patients had no known risk factor. The majority of them presented with complaints of facial swelling (n=16, 37.2%) and nasal blockage (n=11, 25.6%). Characteristic features and demographics of the included patients are shown in Table 1.

**Table 1:** Demographic and patient characteristic features.

Patient Characteristics	Frequency (n)	Percentage (%)
Gender		
Male	26	60.5%
Female	17	39.5%
T Stage		
1	0	0%
2	1	2.3%
3	4	9.3%
4	36	83.7%
X	2	4.7%
N Stage		
N0	36	83.7%
N1	5	11.6%
N2	2	4.7%
Overall stage		
I	0	0%
II	1	2.3%
III	4	9.3%
IV	36	83.7%
X	2	4.7%
Presenting symptoms		
Facial swelling	16	37.2%
Nasal obstruction	11	25.6%
Epistaxis	8	18.6%
Visual disturbance	1	2.3%
Neurological deficit	2	4.6%
Others	5	11.6%
Risk factors		
Smoking	13	30.2%
Tobacco chewing	7	16.3%
Betel nuts	5	11.6%
Alcohol	2	4.6%

The American Joint Committee on Cancer (AJCC) staging system was used for staging before treatment [17]. Out of 43 patients, 36 (83.7%) patients had stage IV disease, and cervical node metastasis was present in 7 (16.3%) patients (Table 1).

All the cases were discussed on the multidisciplinary tumor (MDT) board. Surgery was performed in 16 (37.2%) patients; 4 (9.3%) patients out of these received induction chemotherapy preoperatively due to advanced disease and borderline operability, whereas the remaining 27 (62.8%) patients received chemoradiation due to advanced stage and inoperable disease. Amongst the 16 patients planned for surgery, total maxillectomy was performed in 5 (11.6%) while partial maxillectomy was done in 11 (25.6%) cases. Six (13.9%) patients underwent therapeutic neck dissection for cervical metastasis.

**Figure 1:**
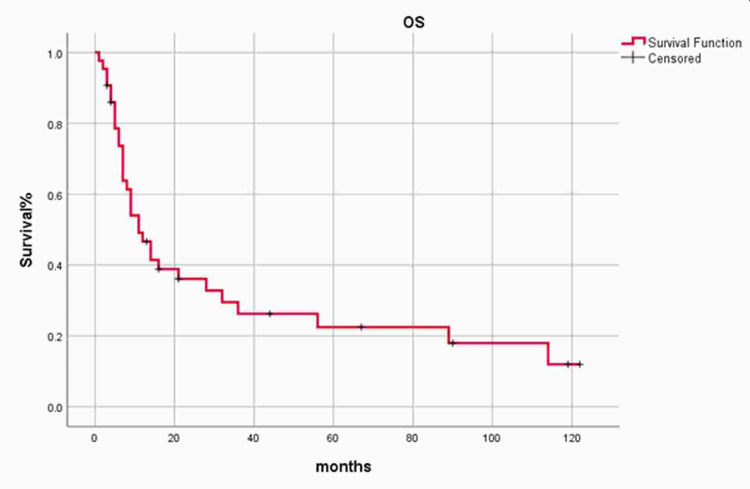
Overall survival of Squamous Cell Carcinoma of Maxillary Sinus

All patients were followed postoperatively, and the mean follow-up time was 49.5 months (SD ± 11.8). Failure of treatment was seen in 35 (81.4%) patients, which included 30 cases (85.7%) with locoregional disease recurrence and 5 (14.3%) with distant metastases. Based on the likelihood ratio Chi-square test, treatment failure was not significantly associated with the size of the tumor, nodal status, overall stage of the disease, and treatment modalities (Table 2). Our study's five-year overall survival was 22% (Figure 1).

**Table 2:** Association with treatment failure. P-value is taken as significant if it is ≤0.05

Characteristics	Patients with no recurrence	Locoregional recurrence	Distant metastasis	P-Value
T Stage				0.175
1	0	0	0	
2	0	1	0	
3	2	1	1	
4	6	27	3	
X	0	1	1	
Nodal status				0.831
Positive	3	2	2	
Negative	5	28	3	

**Figure 2:**
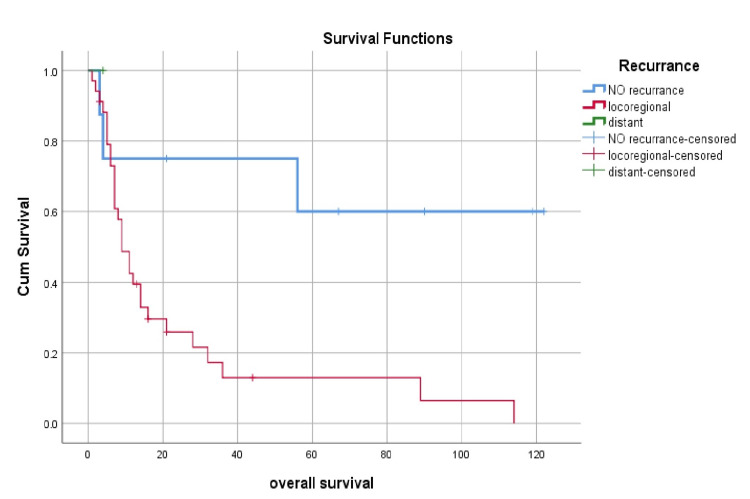
Survival according to the pattern of treatment failure

**Figure 3:**
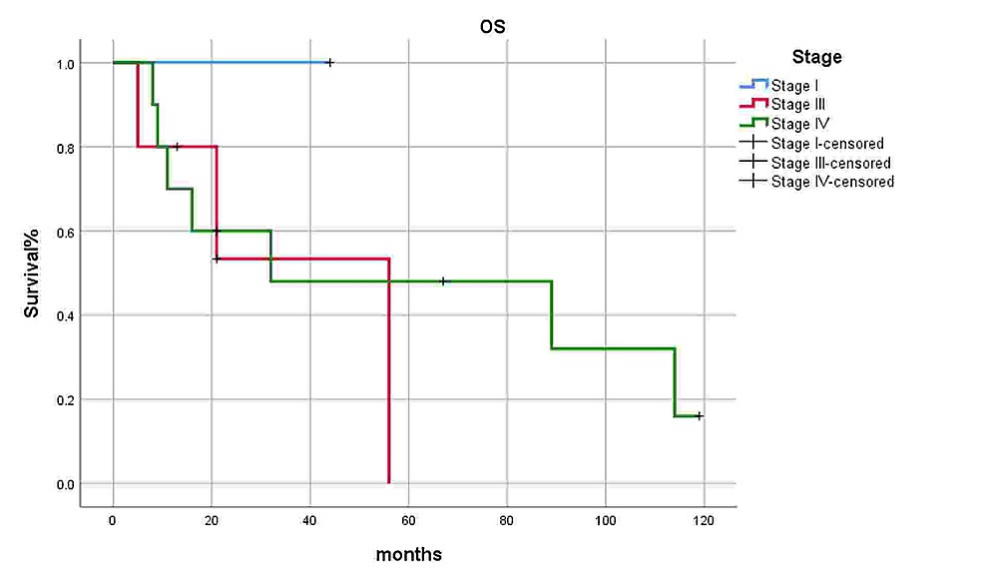
Survival according to overall staging.

Loco-regional recurrence and distant metastasis were significant factors impacting survival (p=0.021), while age, gender, staging, and treatment modalities have not affected survival outcomes (Figures 2, 3). Kaplan Meier curves present survival, and the p-value is calculated using the log-rank test.

## Discussion

Maxillary sinus SCC is one of the aggressive malignancies with a relatively poor prognosis [18]. Malignancies of the nasal cavity and paranasal sinuses are rare, maxillary sinus being the most common subsite accounting for approximately 80% of the malignant tumors arising from it [19]. There is insufficient data on the maxillary sinus SCC; therefore, the incidence is usually measured from independent, institution-based literature and meta-analyses with a limited number of patients [20,21]. The literature shows that the overall survival outcomes of maxillary sinus SCC are still not promising despite the advancement in the development of diagnosis and treatment strategies [22]. Different patterns have been reported in the literature based on the experience of independent institutions and retrospective analysis, with various treatment outcomes [23,24].

The tumor can be resected endoscopically if it presents at an earlier stage I and in a few cases with stage II. Delayed presentation at the advanced stage and the proximity of vital structures such as the eye, skull base, infratemporal fossa, and pterygomaxillary fossa make it more challenging to take negative surgical margins. Consequently, for advanced stages, an open approach is required [1].

AJCC staging 8th was applied to stage these tumors in our study. Clinically, most patients remain asymptomatic or present with vague symptoms at an earlier stage until the disease progresses to an advanced stage. Furthermore, the critical factor for delayed presentation and diagnosis is the variety of symptoms caused by this malignancy. As a result, ninety-three percent of our patients presented at an advanced stage. Current literature reports that most of the patients presented at an advanced stage with large tumors involving bones and extension to surrounding vital structures like orbit [25]. The treatment strategy for all the resectable maxillary sinus SCC is surgery followed by adjuvant chemo-radiotherapy [26].

In contrast to other head and neck malignancies, where cervical lymph node metastasis is high, the global data in publications shows that nodal metastasis is rare in Sinonasal malignancies [1,2,27]. Our study noted similar results, where eighty-four percent were lymph node-negative. Despite the low incidence of cervical nodal metastasis in maxillary sinus squamous cell carcinoma, which is managed conservatively, Scurry et al. have reported regional recurrence up to 18% and emphasized the need to perform an elective neck dissection in N0 necks [28].

Literature shows that surgery followed by adjuvant radiotherapy is the more common modality of treatment in the maxillary sinus SCC [29-32]. Surgery-based treatment is more beneficial than systemic therapy [33]. However, Kuo and colleagues described that the overall survival rate of maxillary sinus squamous cell carcinoma patients improved with neoadjuvant treatment [34]. The adjuvant chemo-radiotherapy has a significant role in treating those maxillary sinus tumors with positive margins and adverse features [35,36]. Our study did not notice any significant difference in the survival outcomes based on the treatment modalities.

Local recurrence has proven to be the leading cause of treatment failure compared to distant metastasis [1,2,37]. Park et al. reported a high local recurrence rate after surgery and radiotherapy treatment [38]. In our study, failure of treatment was seen in n=35 (81.4%) cases, with locoregional recurrence in 30 (85.7%) cases and distant metastases in 5 (14.3%) patients.

In literature, the overall 5-year survival rate of maxillary sinus SCC has been reported as 25-50% [2], and our study showed similar results with a 5-year survival rate up to 22%. In our study, locoregional recurrence and distant metastasis significantly affect survival (P=0.001), which is comparable with global data [1]. In our study stage, margins status and treatment modalities were independent factors not significantly associated with treatment failure. However, in literature, evidence shows that postoperative adjuvant radiotherapy or chemotherapy is associated with better survival outcomes in maxillary sinus malignant tumors than surgery alone [32,36,39].

We acknowledge some shortcomings in our study. Ours was a single-center, retrospective study with a limited number of cases to depict survival outcomes. Many studies report the survival rate of sinonasal malignancies with multiple pathologies. It would be desirable to investigate the outcomes and prognostic factors affecting each pathology separately for future publications.

## Conclusions

To summarize, SCC of the maxillary sinus is rare cancer with a higher incidence in males, commonly diagnosed at an advanced stage, and is associated with a poor survival outcome. These tumors have a poor prognosis and are most resistant to treatment. Locoregional recurrence along with distant metastasis are factors that adversely impact the overall survival figure. In contrast, tumor size, nodal status, overall stage, and treatment modalities do not significantly differ.
